# The visibility of the periventricular crossroads of pathways in preterm infants as a predictor of neurological outcome and occurrence of neonatal epileptic seizures

**DOI:** 10.3325/cmj.2021.62.165

**Published:** 2021-04

**Authors:** Branka Bunoza, Nina Barišić, Petra Grđan Stevanović, Ana Bogdanić, Vesna Benjak, Ruža Grizelj, Daniel Turudić, Danko Milošević, Marko Radoš

**Affiliations:** 1 Department of Pediatrics, Division of Pediatric Neurology, University Hospital Center Zagreb, Zagreb, Croatia; 2 University of Zagreb School of Medicine, Zagreb, Croatia; 3 Department of Pediatrics, University Hospital Center Zagreb, Zagreb, Croatia; 4 General Hospital Zabok and Hospital of Croatian Veterans, Zabok, Croatia; 5 Croatian Institute for Brain Research, University of Zagreb School of Medicine, Zagreb, Croatia

## Abstract

**Aim:**

To evaluate the relationship between the neurological outcome, neonatal epileptic seizures, and signal-intensity visibility of the frontal and parietal periventricular crossroads of pathways on brain magnetic resonance imaging (MRI) in preterm infants at term-equivalent age.

**Methods:**

The study enrolled 48 preterm infants born between 2012 and 2016. The signal-intensity characteristics of the frontal and parietal periventricular crossroads were evaluated and classified into four grades. A non-favorable outcome was defined as a motor and functional disorder with developmental delay and/or cerebral palsy.

**Results:**

Neonatal seizures, epilepsy, pathological EEG and brain ultrasound finding, and brain MRI abnormalities were mostly found in neonates with non-favorable outcomes. Visible frontal and parietal periventricular crossroads were associated with a normal neurologic outcome (*P* = 0.0004; *P* = 0.0009, respectively). Not-visible or slightly visible periventricular crossroads were associated with non-favorable outcomes in the case of frontal crossroads (*P* = 0.036) and not-visible periventricular crossroads in the case of both frontal and parietal crossroads (*P* = 0.001, *P* = 0.015, respectively). The visibility of the frontal and parietal periventricular crossroads was associated with a lack of neonatal epileptic seizures (*P* = 0.03; *P* = 0.02, respectively). The frontal crossroads were more frequently slightly visible, while the parietal periventricular crossroads were more frequently visible.

**Conclusion:**

Poor visibility of the frontal and parietal crossroads of pathways on MRI is associated with neonatal epileptic seizures and poor neurological outcomes in preterm infants at term-equivalent age.

The advancement of neonatal medicine has increased the survival rates of preterm children with neonatal white matter brain injury and a higher risk of poor developmental outcomes, including motor and cognitive deficits with impaired social development. Approximately 10% of preterm neonates (PN) develop cerebral palsy, while 40% experience cognitive loss, language development problems, and a consequent lower educational attainment (1-3). The prevalence of neurodevelopmental difficulties in PNs is higher compared with full-term infants and increases with lower gestational age (4,5).

The neonatal brain MRI is an essential clinical tool for predicting the long-term outcome of brain injury in PN (6,7). However, a significant number of PNs have neurodevelopmental difficulties without having brain abnormalities structurally visible on MRI. A biological substrate of these disabilities could be abnormal brain maturation. The level of cerebral maturity in PNs is estimated by assessing the MRI characteristics of transient fetal compartments (periventricular crossroads areas, subplate, and von Monakow segments) that persist at term-equivalent age (8). According to von Monakow's division of the cerebral white matter into five compartments, the periventricular crossroads of pathways are part of the segment II (9). These transient fetal compartments are where intense developmental processes take place during the second half of gestation, and they are possibly unrecognized cellular and topographic areas affected by perinatal brain lesions.

The periventricular crossroads are located in fetal white matter along the lateral ventricles and correspond to the cross-section of the main cortical projection, associative, and commissural fibers. These areas, characterized by a hydrophilic extracellular matrix, can be well recognized on T2w MRI (10). We hypothesized that the imaging characteristics of these intersections on brain MRI were the biomarkers of neurodevelopmental outcomes. The original study aim was to assess the relationship between the neurological outcome and neuroimaging characteristics. As we obtained encouraging results regarding brain crossroads, we decided to retrospectively review standard T2w MRI brain scan to specifically assess these brain structures. Accordingly, we analyzed the relationship between the visibility of the frontal and parietal crossroads and the occurrence of neonatal epileptic seizures and neurological outcomes.

## Materials and methods

Among PNs without comorbidities associated with preterm birth treated in our Center between 2012 and 2016, we randomly selected 64. The study finally enrolled 48 PNs, as 16 PNs were not included due to brain MRI that was not performed at exact term-equivalent age and chromosomal/other genetic disorders or disorders that may have affected the study results (other neurologic, metabolic, infective diseases).

The children were followed-up by a neuropediatrician every 6 months during 3-7 years until 2019. We collected the data on the gestational age (GA), birth weight, first- and fifth-minute Apgar score, sex, mode of delivery (vaginal delivery or cesarean section), mechanical ventilation, and the occurrence of neonatal seizures. Neonatal seizures were classified according to International League Against Epilepsy (ILAE) criteria (11,12). Inclusion criteria for the neonatal epileptic seizures were established by clinical observation and by more than one conventional EEG recording. We used bipolar EEG recording with conventional 10-20 montage modified for neonates. Epilepsy was diagnosed according to established ILAE criteria (2017) (11). EEGs were classified as normal, moderately abnormal, and severely abnormal.

All PNs underwent serial ultrasound brain scans in the first 24 h, at first, second, and third week, and after 3 months. These findings were classified as 1) normal/moderately abnormal (no abnormalities, or intracranial hemorrhage degree I or II); 2) moderately/severely abnormal (intracranial hemorrhage degree III or IV, intraparenchymal hemorrhage, or cystic periventricular leukomalacia).

**Figure 1:**
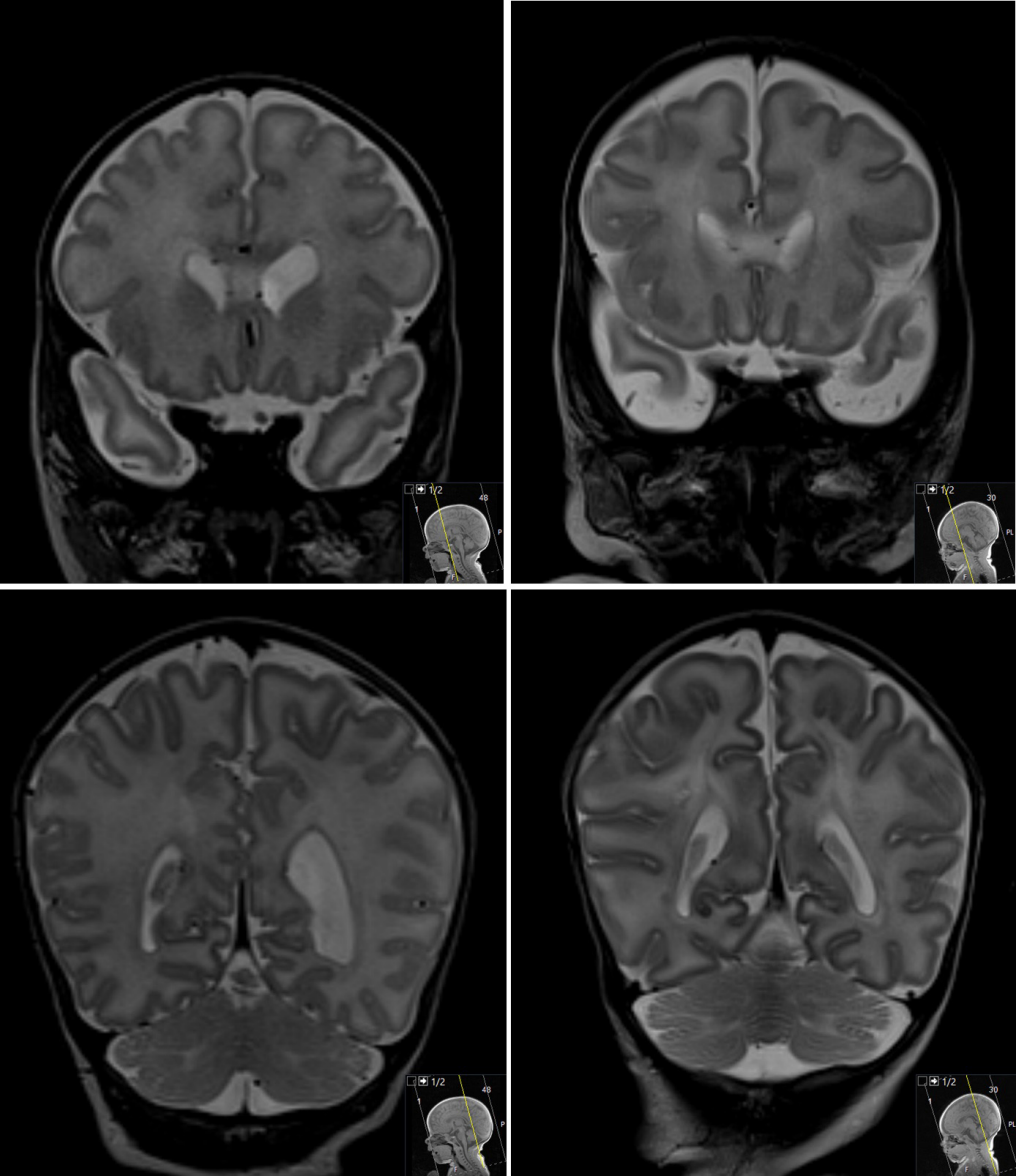
The visibility grades of the periventricular crossroads of pathways: not visible (grade 0), slightly visible (grade 1), visible (grade 2), and clearly visible (grade 3). (**A**) Frontal crossroads area. Left image: grade 3. Right image: grade 0. (**B**) Parietal crossroads area. Left image: grade 2. Right image: grade 1.

Brain MRI was performed at the exact term-equivalent age (40 ± 2 weeks gestational age) with a 3T MRI scanner (Magnetom TrioTim, Siemens, Erlangen, Germany). Coronal T2-weighted turbo spin-echo sequence (TR/TE = 6820/88 ms, flip angle = 120°, field of view = 200 × 140, acquired matrix = 320 × 268, voxel size = 0.7 × 0.6 × 1.2 mm) was used as a standard neuroimaging procedure. The MRI images were obtained during the newborn's postprandial sleep with the use of sedation (phenobarbitone). A neuroradiologist blinded to the clinical data evaluated the visibility of the frontal and parietal periventricular crossroads. The evaluation was based on the histological and MRI descriptions of the transient fetal structures (10,13,14). T2-weighted MRI periventricular crossroads are positively recognized with high MR signal intensity from 24 to 40 weeks GA. Grading was based on the visibility of signal intensity and classified as follows: not visible (grade 0), slightly visible (grade 1), visible (grade 2), and clearly visible (grade 3) (Figure 1).

The neurological outcome was assessed at the age of three years. Neuromotor assessment was performed according to a standardized pediatric neurologic evaluation based on Amiel-Tison and Touwen neurological examination. Cerebral palsy was diagnosed at the age of four years (15). The favorable outcome was classified as normal neurological development or mild muscle tone and reflexes abnormalities, whereas the non-favorable outcome was classified as significant motor and functional abnormalities with a developmental delay or cerebral palsy.

The study was undertaken according to the principles of the Declaration of Helsinki and approved by the Institutional Review Board of the University of Zagreb School of Medicine. The parents provided written informed consent for the children to undergo the study procedures.

### Statistical analysis

Descriptive statistics are reported as frequencies with percentages for categorical variables and as mean, maximum, and minimum value for continuous variables. The Shapiro-Wilk test was used for normality testing. Nominal data were analyzed with the χ^2^ test. Ordinal data analysis and comparison of means of continuous variables were performed with the Kruskal-Wallis one-way analysis of variance and Mann-Whitney U tests. The Kaplan-Meier survival analysis and log-rank test (embedded within the Kaplan-Meier estimator) were performed. The level of significance was set at *P* < 0.05. The analysis was performed with SPSS, version 20 (IBM Corp., Armonk, NY, USA) and STATISTICA, version 13.5 (TIBCO, Palo Alto, CA, USA).

## Results

The mean GA was 29.21 weeks (range 24.00-36.00, SD ±3.031), with almost a half of the infants having been born before 28 weeks GA. The mean birth weight was 1317 g (range 640-2260, SD ±414.682). Due to the immaturity of the children, first-minute (mean 5.8, range 0-10, SD ±2.835) and fifth-minute (mean 7.2, range 6.2-10, SD ±2.275) Apgar scores were low. The number of male and female participants was not significantly different (M:F = 23:25). The majority of PNs were born by a cesarean section (11 or 29.2%), 26 (54.2%) needed resuscitation, and 35 (72.9%) needed mechanical ventilation.

**Figure 2:**
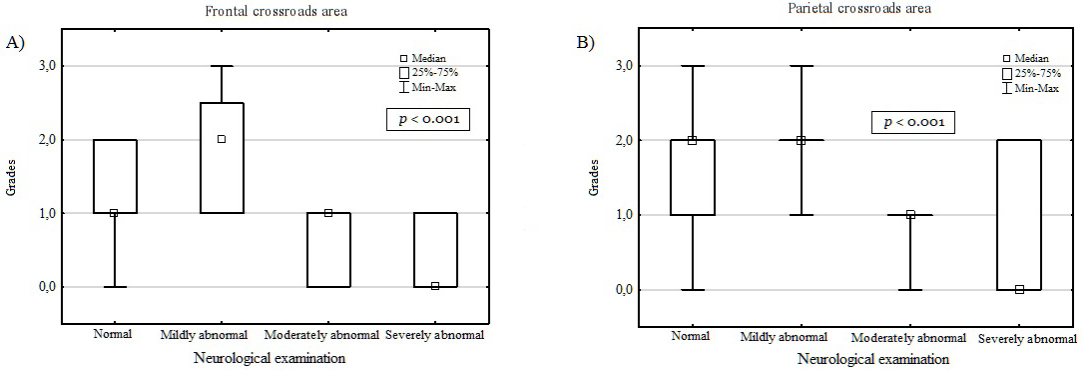
The association of the visibility grades of the periventricular crossroads of pathways and the neurologic outcome (Kruskal-Wallis test). Children with normal/mildly abnormal neurological examination had visibility grades 1-2. In contrast, children with moderately/severely abnormal neurological examination had visibility grades 0-1. (**A**) Frontal crossroads area. (**B**) Parietal crossroads area.

**Figure 3:**
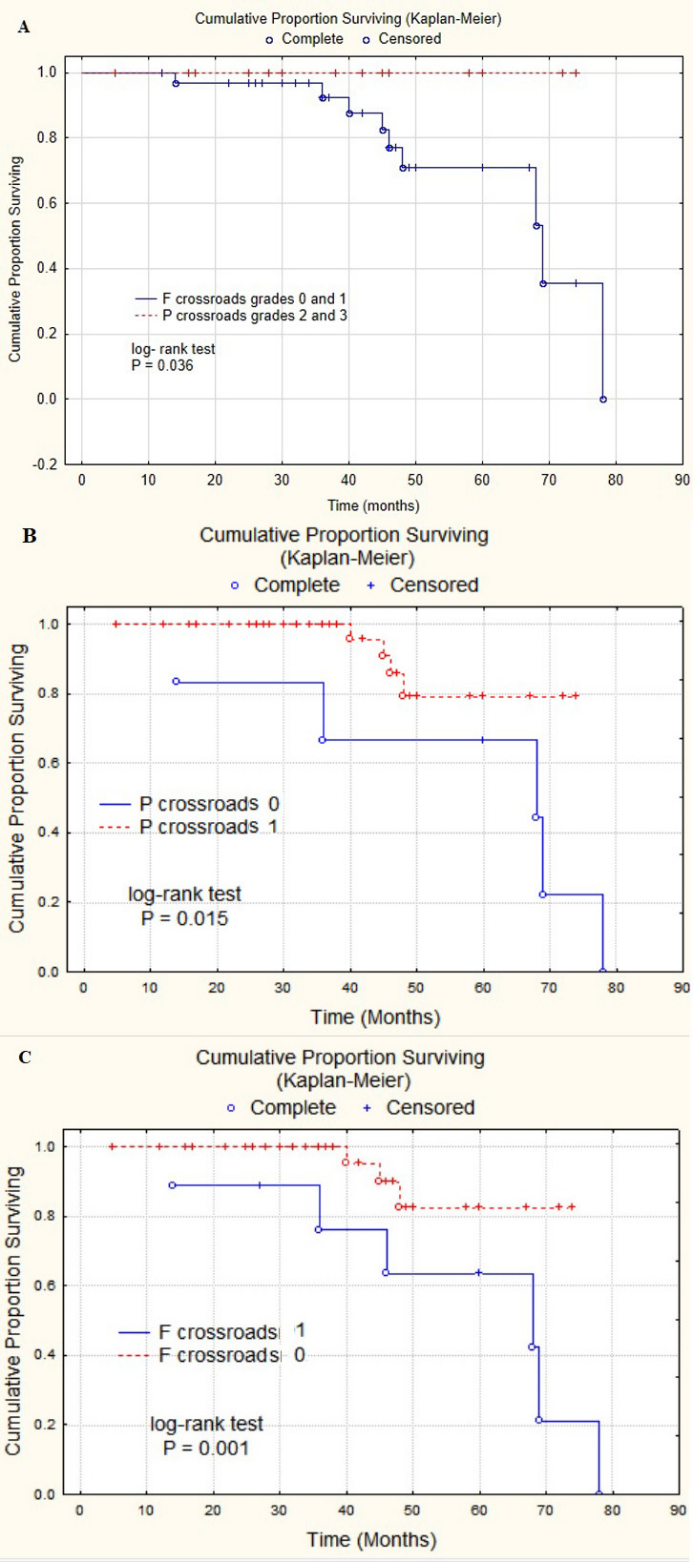
Differences between the visibility grades 0 and 1 vs visibility grades 1 and 2 of the periventricular crossroads of pathways in the frontal crossroads area (**A**). Differences between visibility grade 0 vs grades 1-3 in the parietal (**B**) and frontal crossroads areas (**C**) (log-rank, Kaplan-Meier). Not-visible or slightly visible periventricular crossroads were associated with non-favorable outcomes for frontal crossroads and not-visible for both frontal and parietal crossroads. F – frontal; P – parietal; 0 – not visible;1 – slightly visible; 2 – visible; 3 – clearly visible; 0 = F/O; 1 = N/O.

**Figure 4:**
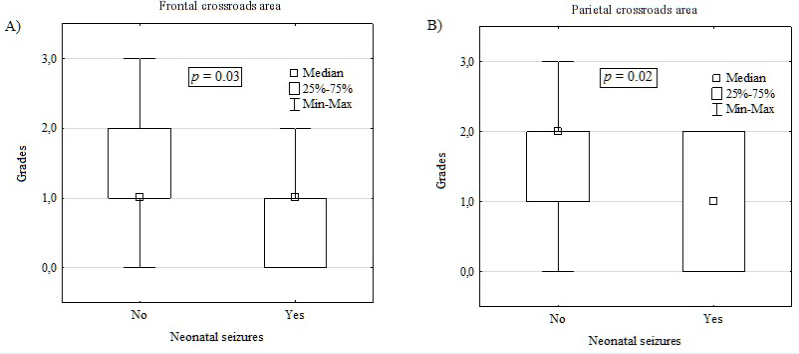
The visibility grades of the periventricular crossroads of pathways according to the occurrence of epileptic seizures in the neonatal period (Mann-Whitney U test). Children with epileptic seizures had periventricular crossroads of pathways visibility grade 1, whereas children without epileptic seizures had visibility grades 1-2. (**A**) Frontal crossroads area. (**B**) Parietal crossroads area.

The average follow-up lasted 46 months (median). Favorable outcomes were identified in 39 children. Neonatal seizures, epilepsy, pathological EEG finding, brain ultrasound, and brain MRI abnormalities were found mostly in the non-favorable outcome group. The groups did not differ in the mode of delivery, sex, gestational age, birth weight, and Apgar score. The MRI brain scans were classified according to the frontal and parietal periventricular crossroads visibility grades. Visible and clearly visible crossroads (visibility grades 2 and 3) in both frontal and parietal areas were associated with favorable outcomes, while non-visible and slightly visible (visibility grades 1 and 2) crossroads were associated with non-favorable outcomes. None of the PNs with visibility grade 3 had a non-favorable outcome, whereas a significantly higher percentage of PNs with visibility grades 1 and 2 had poor neurological outcomes (Table 1). PNs with normal/mildly abnormal neurologic outcome had a better visibility, while PNs with moderately/severely abnormal neurological outcomes a had poor visibility of the frontal and parietal periventricular crossroads (*P* = 0.004; *P* = 0.0009, respectively) (Figure 2A and B). The log-rank test was used to assess the differences between frontal and parietal visibility grades 0 and 1 vs visibility grades 2 and 3. Visibility grades 0 and 1 were significantly associated with non-favorable outcomes for the frontal (*P* = 0.036) but not for the parietal crossroads (*P* = 0.067) (Figure 3A). As the group of PNs with visibility grade 0 consisted of a considerable proportion of children with non-favorable outcomes, additional tests were performed comparing the crossroads visibility grade 0 and crossroads visibility grades 1-3. Visibility grade 0 was significantly associated with non-favorable outcomes for both the frontal and parietal crossroads (*P* = 0.001 vs *P* = 0.015, respectively) (Figure 3B and 3C). The crossroads visibility was significantly negatively related to the occurrence of neonatal epileptic seizures (*P* = 0.03; *P* = 0.02, respectively). The PNs without neonatal seizures had a better visibility of the frontal and parietal periventricular crossroads (Figure 4 A and B).

**Table 1:** Clinical characteristics of outcomes in preterm neonates (N = 48)

	Patients with favorable outcomes (n = 39)		Patients with non-favorable outcomes (n = 9)	P
**Neonatal seizures**				
no	33		4	0.010
yes	6		5	
**Epilepsy**				
no		38	6	0.003
yes		1	3	
**EEG**				
normal	22		2	0.016
moderately abnormal	12		2	
severely abnormal	5		5	
**Ultrasound brain scan**				
normal/mildly abnormal	28		0	<0.001
moderately/severely abnormal	11		9	
**Magnetic resonance imaging brain scans**				
normal	18		0	<0.001
mildly abnormal	8		0	
moderately abnormal	13		5	
severely abnormal	0		4	
**Frontal crossroads**				
invisible	1		5	<0.001
mildly visible	11		2	
moderately visible	22		2	
clearly visible	5		0	
**Parietal crossroads**				
invisible	3		6	<0.001
mildly visible	20		3	
moderately visible	13		0	
clearly visible	3		0	

**Figure 5:**
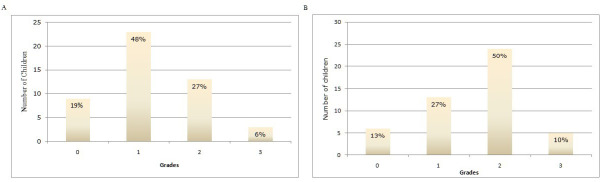
Visibility grades of frontal and parietal periventricular crossroads of pathways. Grade 1 visibility was predominant in the frontal (**A**), and grade 2 in the parietal crossroads area (**B**).

Differences between frontal and parietal crossroads visibility grades were observed as well. Grade 1 visibility predominated in the frontal, and grade 2 visibility predominated in the parietal periventricular crossroads of pathways (Figure 5 A and B).

## Discussion

In this study, we showed an association between not visible/slightly visible frontal and parietal crossroads of pathways on MRI and an unfavorable neurodevelopmental outcome. After perinatal injury in PNs it is essential to predict the neurodevelopmental outcome by a neonatal MRI scoring system (16). We described transient cerebral segments of white matter according to von Monakow and recent studies based on histological and MRI analyses (9,10).

The visibility and pattern of brain transient fetal compartments on T2 weighted MRI that persist to term age in newborns were previously suggested as markers of neurodevelopmental outcome (8). A strong association with the neurological outcome also makes the crossroads' visibility a sign of normal or poor development. Previous studies showed that clearly visible crossroads were a predictor of normal neurodevelopment, and that the extent of signal-intensity characteristics of the periventricular crossroads in the frontal and parietal regions could be graded (17-20). Our study complemented this knowledge by finding that unfavorable neurodevelopmental outcome was associated with the visibility of both the parietal and frontal crossroads of pathways, even more so with that of the frontal ones. A previous study assessing the correlation of MR imaging with histochemical results recognized that the early preterm periventricular crossroads contain hydrophilic extracellular matrix with axonal guidance molecules, which are easily recognized as hypointensities on T1-weighted MR images or hyperintensities on T2-weighted MR images (10). These periventricular crossroads regions are histologically detectable from the 20th week of gestation, and from the 24th week on fetal MR imaging. In many infants, they remain visible up to the term-age (21).

Therefore, perinatal white matter lesions and periventricular white matter crossroads may represent an unrecognized and vulnerable cellular and topographic target, in which a combined damage to associative-commissural and projective fibers may explain cognitive, sensory, and motor deficit. The periventricular crossroads areas may be wrongly interpreted as periventricular leukomalacia, because of similar MR imaging signal intensity features (10). As some inborn metabolism errors and perinatal hypoxia could have a similar high signal intensity on MRI imaging with an abnormal white matter signal, our study did not include PNs with metabolic disorders.

Most PNs had epileptic seizures during the first 48 hours of life, with multiple types of seizures with variable features (clonic, tonic, subclonal/electrographic). However, the underlying causes of seizures, rather than the types of seizures *per se*, have been considered critical determinants of neurological abnormalities later in life (22,23). In our study, the clearly visible/visible frontal and parietal periventricular crossroads were associated with a lack of epileptic seizures, while non-visible/slightly visible periventricular crossroads areas were associated with a poor neurodevelopment outcome.

Visibility differences between the frontal and parietal crossroads of pathways and decreased visibility in the frontal area are difficult to explain. We agree with the assumption that poorly visible parietal crossroads of pathways are associated with a poor outcome in PNs (24). Lower visibility of the frontal compared with parietal crossroads indicates their higher sensitivity to perinatal injury and predicts a non-favorable outcome with neurological damage. Damage to the frontal crossroads is likely to be more important for poor neurological prognosis than damage to the parietal crossroads. The association of seizure types and neurological outcome with frontal/parietal crossroads remains to be determined.

The study limitations are a small sample size and a relatively short follow-up period. Continuous amplitude-integrated EEG was not routinely performed and PNs with a severe cerebral injury caused by prematurity (including cystic periventricular leukomalacia) were not excluded from the study. A further study with a larger number of PNs is warranted.

In conclusion, we believe that neonatal visibility of the periventricular crossroads of pathways has a predictive value for the neurological outcome. Crossroads' characteristics on T2w MRI at term-equivalent age could serve as biomarkers of brain injury.
